# The importance of involving healthcare professionals in the production of neurodiversity healthcare training

**DOI:** 10.1192/j.eurpsy.2023.77

**Published:** 2023-07-19

**Authors:** B. French

**Affiliations:** University of Nottingham, Nottingham, United Kingdom

## Abstract

**Abstract:**

Attention deficit hyperactivity disorder (ADHD) is underdiagnosed in the UK and the assessment and diagnosis pathway involves multiple healthcare professionals, often starting with a general practitioner (GP) referral to specialist services. GPs’ levels of knowledge and understanding about ADHD is often a significant barrier in patients accessing care. Better understanding of ADHD is needed.

**Method:**

A step wise, co-production approach towards developing an online ADHD education intervention for GPs was followed. Preparatory work highlighted the relevant topics to be included in the intervention and workshops were then conducted with GPs, leading to further refinement of the content and the final intervention. A pilot usability study (n= 10 GPs) was conducted to assess the intervention’s acceptability and feasibility, followed by a randomised controlled trial (n= 221 GPs) to assess its efficacy and impact on knowledge and practice.

**Results:**

The development of the online intervention was greatly facilitated by the involvement of GPs. Having a co-production development process ensured the consistent adaptation of the intervention to meet GPs’ needs. The usability study showed that the content of the intervention was suitable, easily accessible, engaging and delivered at an acceptable level of intensity, validating the development approach taken. The knowledge (P<.001) and confidence (P<.001) of the GPs increased after the intervention, whereas misconceptions decreased (P=.04); this was maintained at the 2-week follow-up. Interviews and surveys also confirmed a change in practice over time

**Conclusion:**

This project highlights the importance of co-development in developing educational program that addresses specific needs for GPs. Involving end-users in co-creating interventions enhances their clinical utility and impacts routine clinical practice

**Disclosure of Interest:**

None Declared

